# Periostin/Bone Morphogenetic Protein 1 axis axis regulates proliferation and osteogenic differentiation of sutured mesenchymal stem cells and affects coronal suture closure in the TWIST1^+/−^ mouse model of craniosynostosis

**DOI:** 10.1186/s13018-024-04604-3

**Published:** 2024-02-19

**Authors:** ShuBin Feng, Qiang Feng, LiuJian Dong, Qiang Lv, ShiYue Mei, YaoDong Zhang

**Affiliations:** 1 Department of Neurosurgery, Children’s Hospital Affiliated of Zhengzhou University, Henan Children’s Hospital, Zhengzhou City, 450018 Henan Province China; 2 Henan Key Laboratory of Children’s Genetics and Metabolic Diseases, Children’s Hospital Affiliated of Zhengzhou University, Henan Children’s Hospital, No.33, Longhu Outer Ring East Road, Zhengdong New District, Zhengzhou City, 450018 Henan Province China

**Keywords:** Craniosynostosis, Periostin, BMP1, Coronal suture closure, Osteogenesis

## Abstract

**Background and objective:**

The pathogenesis of coronal suture craniosynostosis is often attributed to the dysregulated cellular dynamics, particularly the excessive proliferation and abnormal osteogenic differentiation of suture cells. Despite its clinical significance, the molecular mechanims of this condition remain inadequately understood. This study is dedicated to exploring the influence of the Periostin/Bone Morphogenetic Protein 1 (BMP1) axis on the growth and osteogenic maturation of Suture Mesenchymal Stem Cells (SMSCs), which are pivotal in suture homeostasis.

**Methods:**

Neonatal TWIST Basic Helix-Loop-Helix Transcription Factor 1 heterozygous (TWIST1^+/−^) mice, aged one day, were subjected to adenoviral vector-mediated Periostin upregulation. To modulate Periostin/BMP1 levels in SMSCs, we employed siRNA and pcDNA 3.1 vectors. Histological and molecular characterizations, including hematoxylin and eosin staining, Western blot, and immunohistochemistry were employed to study suture closure phenotypes and protein expression patterns. Cellular assays, encompassing colony formation, 5-ethynyl-2'deoxyuridine, and wound healing tests were conducted to analyze SMSC proliferation and migration. Osteogenic differentiation was quantified using Alkaline Phosphatase (ALP) and Alizarin Red S (ARS) staining, while protein markers of proliferation and differentiation were evaluated by Western blotting. The direct interaction between Periostin and BMP1 was validated through co-immunoprecipitation assays.

**Results:**

In the TWIST1^+/−^ model, an upregulation of Periostin coupled with a downregulation of BMP1 was observed. Augmenting Periostin expression mitigated craniosynostosis. In vitro, overexpression of Periostin or BMP1 knockdown suppressed SMSC proliferation, migration, and osteogenic differentiation. Periostin knockdown manifested an inverse biological impact. Notably, the suppressive influence of Periostin overexpression on SMSCs was effectively counteracted by upregulating BMP1. There was a direct interaction between Periostin and BMP1.

**Conclusion:**

These findings underscore the significance of the Periostin/BMP1 axis in regulating craniosynostosis and SMSC functions, providing new insights into the molecular mechanisms of craniosynostosis and potential targets for therapeutic intervention.

## Introduction

Craniosynostosis is a common congenital skull defect characterized by premature closure of the coronal suture [1]. This disease causes abnormal growth and development of the skull, which can lead to a series of serious problems such as abnormal head shape, brain development limitation, and intellectual development disorders [2]. Some regulatory factors and molecular mechanisms are related to craniosynostosis, including the mutation of the TWIST1 gene. TWIST1^+/−^ mice have been taken as a representative animal model for craniosynostosis [3]. However, the detailed mechanism of TWIST1 gene mutation causing craniosynostosis has not been fully elucidated.

The key to non-surgical targeted treatment of craniosynostosis is to inhibit and prevent the excessive proliferation and abnormal osteogenic differentiation of cranial suture mesenchymal stem cells (SMSCs) [4]. The abnormal proliferation and osteogenic differentiation of SMSCs is a mechanism leading to craniosynostosis [5]. Periostin is an extracellular matrix protein, which is mainly secreted by osteoblasts and precursor cells, and has different functions during bone development and maturation [6]. Periostin can significantly inhibit the proliferation of coronal suture cells to improve craniosynostosis [7].

Bone Morphogenetic Protein 1 (BMP1) is an enzyme belonging to the metalloproteinase family [8]. BMP1 not only participates in bone formation, but also embryonic development, collagen synthesis, and formation of other tissues [9, 10]. During bone formation, BMP1 interacts with other members of the bone-forming protein family and bone matrix proteins to form a complex signaling network [11]. These interactions regulate the proliferation and differentiation of stem cells, regulate the activity of osteocytes, and thus affect bone formation and regeneration [12].

This study aims to further explore the functions and regulatory mechanisms of Periostin and BMP1 in craniosynostosis, analyze the Periostin/BMP1 axis in the proliferation and osteogenic differentiation of SMSCs, and explore its interaction with mutations in the TWIST1 gene.

## Materials and methods

### Animal model

The animal care procedures followed the provisions of the Animal Care and Use Committee of Children's Hospital Affiliated of Zhengzhou University. TWIST1^+/−^ mice and TWIST1^+/+^ mice (male) were obtained from Cyagen Biosciences Inc. (Suzhou, China). All mice were 1 day old. To up-regulate Periostin, the adenovirus overexpression vector was injected into mice through the tail vein (10 μL, 5 × 10^9^ PFU, Sangon Biotech, Shanghai, China) 1 day after birth. After 3 weeks, the mice were exposed to excessive CO_2_, and the skull tissue of 4 mice in each group was preserved in 4% paraformaldehyde, and that of the remaining 4 mice was preserved at -80℃.

### Hematoxylin and eosin (HE) staining

The skull, fixed in 4% paraformaldehyde, was decalcified in 10% ethylenediaminotetraacetate solution (pH 7.4) for 3 weeks, embedded in paraffin, and cut into 5 µm sections. The sections were stained by HE and observed under the microscope (Olympus IX53).

### Immunohistochemistry

After dewaxing and dehydration, the sections were immersed in 3% H_2_O_2_ to inactivate the endogenous enzymes. After treatment with 5% BSA for 20 min, the sections were added with Periostin (ab14041, Abcam) and BMP1 (ab205394, Abcam) at 4 °C overnight, horseradish peroxidase-labeled secondary antibody at 37℃ for 30 min, and streptavidin–biotin complex at 37℃ for 20 min. Following color development using diaminobenzidine, the sections were stained with hematoxylin, dehydrated and permeabilized with xylene, and sealed with neutral balsam.

### Cell culture and sorting

SMSCs were collected [13]. Simply put, the sagittal and coronal sutures of 1-day-old TWIST1^+/−^ and TWIST1^+/+^ mice were carefully excised within 0.5 mm of adjacent bone on both sides under an anatomical microscope (Leica, M60). The periosteum and cerebral dura mater were removed. The sutures were then washed with phosphate-buffered saline (PBS) and alpha-minimum essential medium (α-MEM) (containing 100 U/ml penicillin and 100 μg/ml streptomycin), prepared into small pieces, and transferred to a T25 Petri dish (Nest, 705001). The cell culture medium was made containing α-MEM (Gibco, 2065542), 20% bovine serum (Gibco, 2100184), 2 mM L-glutamine, 55 μM 2-mercaptoethanol (Gibco, 2090354), 100 U/ml penicillin, and 100 μg/ml streptomycin (Gibco, 2019321). After 5–6 days, the cells were digested with TrypLE (Gibco, 1897328), filtered through a 70 μm cell filter (Falcon, 352350), and incubated for another 3–4 days. The cells were then filtered with a 40 μm cell filter (Falcon, 352340), and SMSCs (CD44^+^CD105^+^) were sorted by flow cytometry using FACS Diva software in the FACS Aria system. After sorting, SMSCs were centrifuged for follow-up experiments.

### Cell transfection

siRNA and pcDNA 3.1 overexpression vectors targeting BMP1 and Periostin (RiboBio, Guangzhou, China) were transfected into SMSCs using Lipofectamine 3000 (Invitrogen, USA), and SMSCs were collected 48 h later for evaluating the transfection efficiency by real-time reverse transcriptase-polymerase chain reaction (RT-qPCR) and Western blot.

### Colony formation experiment

SMSCs were grown in 6-well plates at 500 cells per well and cultured for 2 weeks. After PBS washing, SMSCs were fixed with 4% paraformaldehyde and stained with 0.5% crystal violet (V5265, Sigma-Aldrich). Colonies were counted manually (≥ 100 μm in diameter) to reflect cell proliferation capacity.

### 5-ethynyl-2'deoxyuridine (EdU) experiment

SMSC proliferation was measured using an EdU kit (C10310, RiboBio) as recommended by the manufacturer. In short, SMSCs in the 96-well plate were incubated with 100 μl 50 μM EdU at 37 °C, fixed with 4% paraformaldehyde, and stained with DAPI. EdU-positive cells were observed by fluorescence microscopy and analyzed to reflect changes in cell proliferation.

### Wound healing test

SMSCs in the 6-well plate were cultured for 48 h to reach 90% confluence. Wounds were formed on the surface of monolayer cells using a 200 μL pipette tip, and SMSCs were cultured in a serum-free medium and observed under an inverted optical microscope (Axioskop 40, Carl Zeiss AG, Dresden, Germany) at 0 and 24 h. Wound healing rate = (initial width of wound—width of wound after 24 h)/initial width of wound × 100%.

### Osteogenic differentiation

Osteogenic differentiation of SMSCs was induced using osteogenic differentiation medium (Sigma-Aldrich) containing Dulbecco's modified Eagle's medium (DMEM), 10% fetal bovine serum (FBS), 100 IU/ml penicillin, 100 IU/ml streptomycin, 0.1 μM dexamethasone, 10 mM β-glycerol phosphate, 25 μL bone morphogenetic protein, and 50 μM ascorbic acid. The medium was changed every 4 days, and after 14 days the cells were stained with alkaline phosphatase (ALP) and alizarin red S (ARS).

### ALP staining

SMSCs (1 × 10^4^ cells/well) were plated in a 6-well plate. ALP activity was determined using the ALP staining kit (Thermo Fisher Scientific, Inc.). Subsequently, optical density values were measured using a microplate reader (Infinite™ M2000; Tecan Group, Ltd.) at 540 nm, and images were collected under an inverted fluorescence microscope (Olympus).

### ARS staining

SMSCs were cultured at 5 × 10^5^ cells per well in the 24-well plates containing DMEM-10% FBS. SMSCs were washed with PBS, fixed with 10% formaldehyde-calcium solution for 10 min, washed with isopropyl alcohol for 1 min, and stained with ARS (Leagene, Beijing, China) at 37 °C in the dark for 1 min. After decolorization, SMSCs were re-stained with hematoxylin (Abcam) for 1 min, washed with PBS, sealed with glycerin, and viewed under a microscope [14].

### Western blot

The proteins were isolated using radioimmunoprecipitation assay lysis buffer (Thermo Fisher) and quantified using the BCA Kit (BioVision, USA). After separation by 10% sodium dodecyl sulphate–polyacrylamide gel electrophoresis and loading onto polyvinylidene fluoride membranes (Thermo Fisher), the proteins were blocked with 5% milk and incubated with primary antibodies overnight and a secondary antibody conjugated with horseradish peroxidase (BD Biosciences) at room temperature for 1 h. Protein banding was evaluated using the SynGene system and GeneSnap software (SynGene, USA). Primary antibodies: Glyceraldehyde-3-phosphate dehydrogenase (2118, Cell Signaling Technology), Periostin (ab14041, Abcam), BMP1 (ab205394, Abcam), osteopontin (OPN) (AB5405, MilliporeSigma), runt-related transcription factor 2 (RUNX2) (ab23981, Abcam).

### Data analysis

Data were expressed as mean  ± standard deviation (SD). Each experiment was biologically replicated at least three times. Shapiro–Wilk was used for normality tests, Student t-test for two-group comparison, one-way ANOVA for multi-group comparison, and Tukey HSD for post-hoc test. *P* < 0.05 suggested a significant difference.

## Results

### Periostin improves craniosynostosis in mice with TWIST1^+/−^ mutation at the coronal suture

**Fig. 1:**
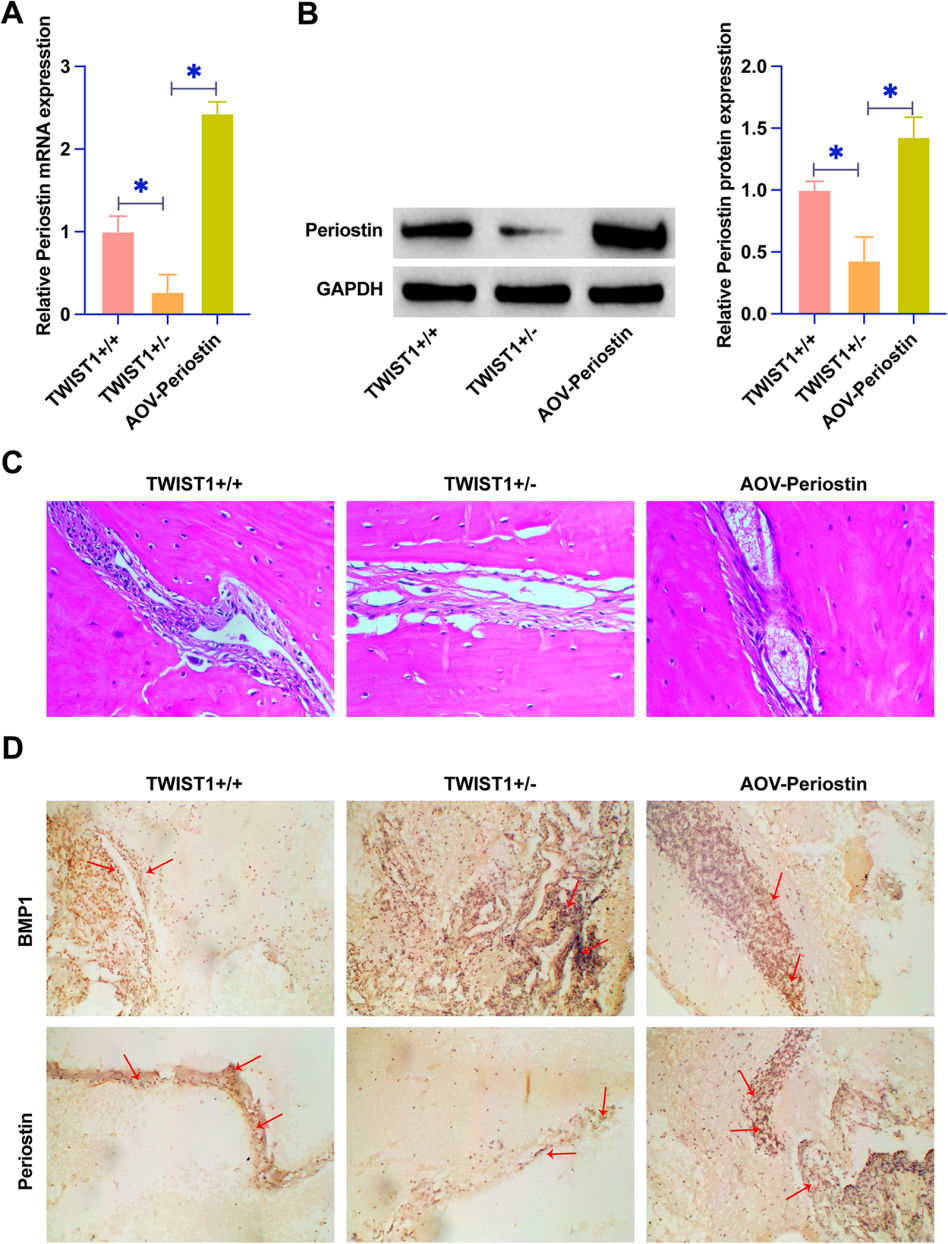
Periostin improves coronal craniosynostosis in TWIST1^+/−^ mice. **A**–**B** RT-qPCR and Western blot measurements of Periostin in mice. **C** HE staining of cranial suture. **D** IHC staining of Periostin and BMP1 in cranial suture of mice. Data were expressed as mean ± SD (n = 4). * *P* < 0.05

In our quest to decode the molecular intricacies behind craniosynostosis in TWIST1^+/−^ mice, we strategically enhanced Periostin expression via targeted adenoviral-mediated overexpression. This intervention was pivotal in unraveling the downregulated expression profile of Periostin in the TWIST1^+/−^ phenotype, as evidenced by RT-qPCR and Western blot. Notably, the upregulation of Periostin enhanced its expression to significant levels (Fig. 1A, B). HE staining illuminated a stark contrast in suture morphology between genotypes. While TWIST1^+/+^ mice displayed an intact coronal suture, their TWIST1^+/−^ counterparts presented with notable membranous adhesion and osteoid deposition. However, these aberrant features were absent in TWIST1^+/−^ mice subjected to Periostin overexpression, underscoring a potential therapeutic effect (Fig. 1C). Immunohistochemistry observed a differential expression pattern at the suture sites: downregulated Periostin and upregulated BMP1 in TWIST1^+/−^ mice compared to the wild-type. Intriguingly, this dysregulated expression pattern was effectively reversed upon Periostin overexpression (Fig. 1D). Collectively, these findings not only highlighted the efficacy of Periostin overexpression in mitigating craniosynostosis in TWIST1^+/−^ mice but also underscored the critical involvement of the Periostin/BMP1 axis in craniosynostosis.

### Periostin effectively inhibits the proliferation and osteogenic differentiation of SMSCs

**Fig. 2:**
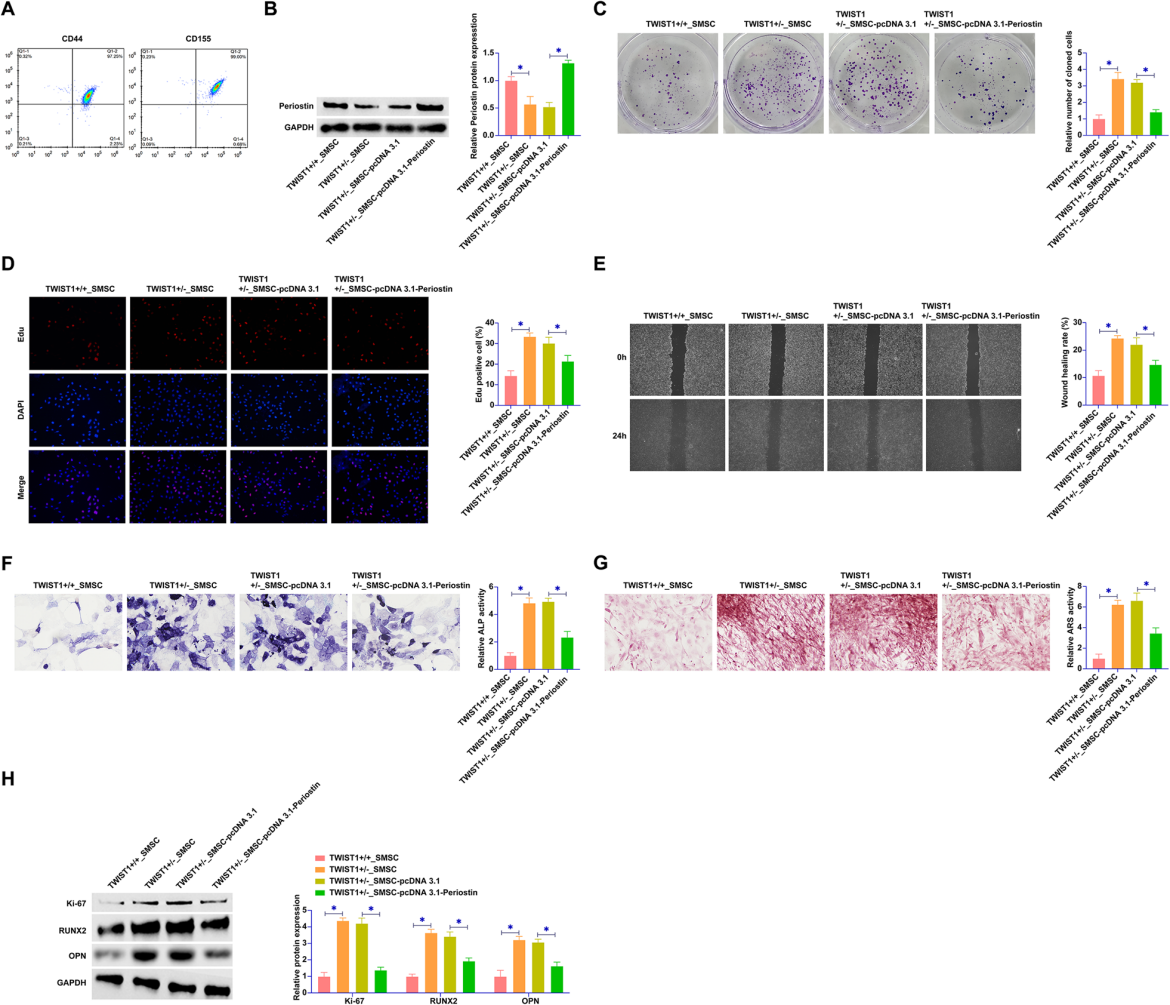
Periostin effectively inhibits SMSC proliferation and osteogenic differentiation. **A** SMSCs were isolated from cranial suture bone tissues of both TWIST1^+/−^ and TWIST1^+/+^ mice and identified by flow cytometry as CD44 and CD105 positive. TWIST1^+/−^ SMSCs were transfected with pcDNA 3.1-Periostin. **B** Western blot assays were performed to assess Periostin protein expression in each group of SMSCs. **C** Colony formation assay was conducted to evaluate the proliferative capacity of SMSCs. **D** EdU assay was used to further quantify SMSC proliferation. **E** Wound healing assays were carried out to measure the wound closure rate of SMSCs. **F** ALP staining was used to determine ALP activity in SMSCs. **G** ARS staining was employed to observe calcium deposition in SMSCs. **H** Western blot analysis was conducted to measure the expression levels of Ki-67, RUNX2, and OPN in SMSCs. Data were presented as mean ± SD (N = 3). *P < 0.05

We isolated SMSCs from the cranial sutures of both TWIST1^+/−^ and TWIST1^+/+^ mice. The cells, positively marked by CD44 and CD105 (Fig. 2A), served as a cellular foundation for subsequent analyses. The transfection of these SMSCs with pcDNA 3.1-Periostin presented a remarkable upregulation in Periostin expression in the TWIST1^+/−^ group, a stark contrast to their baseline underexpression relative to the TWIST1^+/+^ group (Fig. 2B). The impact of Periostin overexpression was assessed through a series of functional assays. Colony formation and EdU assays found a marked suppression in the proliferative capacity of TWIST1^+/−^ SMSCs post-Periostin upregulation (Fig. 2C, D). This trend was further echoed in the wound healing assays, where cellular migration retardation was noted in Periostin-overexpressed TWIST1^+/−^ SMSCs (Fig. 2E). TWIST1^+/−^ SMSCs exhibited a heightened ALP activity and calcium deposition, indicative of an accelerated osteogenic differentiation when compared to their TWIST1^+/+^ counterparts. However, this differentiation process was effectively tempered by overexpression of Periostin (Fig. 2F, G). Western blot probed the expression profiles of key proliferation- and differentiation-related markers. TWIST1^+/−^ SMSCs demonstrated a significantly elevated expression of Ki-67, RUNX2, and OPN. Yet, Periostin overexpression resulted in a downregulation of these markers (Fig. 2H). This suite of data collectively underscored the potent regulatory influence of Periostin overexpression on SMSCs, highlighting its pivotal role in modulating both proliferation and osteogenic differentiation processes within the context of craniosynostosis.

### Knockdown of Periostin promotes the proliferation and osteogenic differentiation of SMSCs

**Fig. 3:**
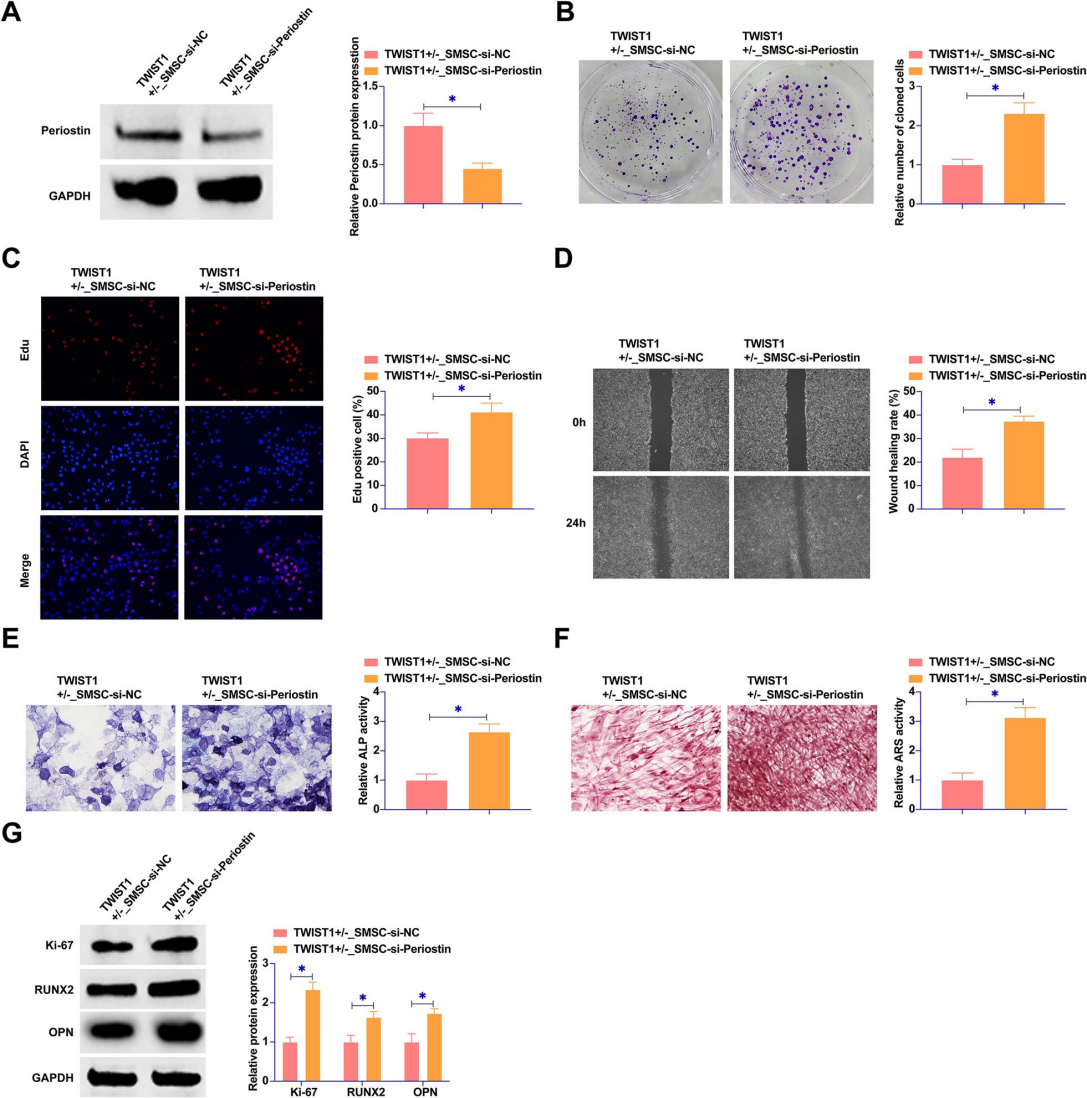
Periostin triggers the proliferation and osteogenic differentiation of SMSCs. si-Periostin was transfected into the SMSCs of the TWIST1^+/−^ group. **A** Western blot assays were used to assess Periostin protein expression in the SMSCs across the different groups. **B** Colony formation assays were conducted to evaluate the proliferative capacity of SMSCs. **C** EdU assays were utilized to further quantify the proliferation of SMSCs. **D** Wound healing assays were carried out to measure the wound closure rate of SMSCs. **E** ALP staining was employed to determine the ALP activity in SMSCs. **F** ARS staining was used to observe calcium deposition in SMSCs. **G** Western blot analysis was conducted to assess the expression levels of Ki-67, RUNX2, and OPN in SMSCs. Data were represented as mean ± SD (N = 3). * *P* < 0.05

The strategic silencing of Periostin in TWIST1^+/−^ SMSCs through si-Periostin transfection marked a significant pivot in our study. This molecular intervention resulted in a decrease in Periostin protein expression (Fig. 3A). The ensuing analyses revealed a notable promotion in SMSC proliferation, as evidenced by increased clonogenic activity and EdU incorporation rates following Periostin knockdown (Fig. 3B, C). This genetic modulation further accelerated wound healing capacity of SMSCs (Fig. 3D). The osteogenic differentiation of SMSCs was significantly promoted after Periostin knockdown, as demonstrated by enhanced ALP activity and calcium deposition (Fig. 3E, F). Additionally, silencing of Periostin upregulated the expression of key cellular markers such as Ki-67, RUNX2, and OPN (Fig. 3G). This upregulation in marker expression reflects a pivotal shift in the cellular behavior of SMSCs, emphasizing the critical role of Periostin in modulating their proliferative and differentiation pathways. In summary, these results underscored the influence of Periostin knockdown on enhancing the proliferative and osteogenic differentiation capabilities of SMSCs.

### Periostin regulates BMP1 expression in SMSCs

**Fig. 4:**
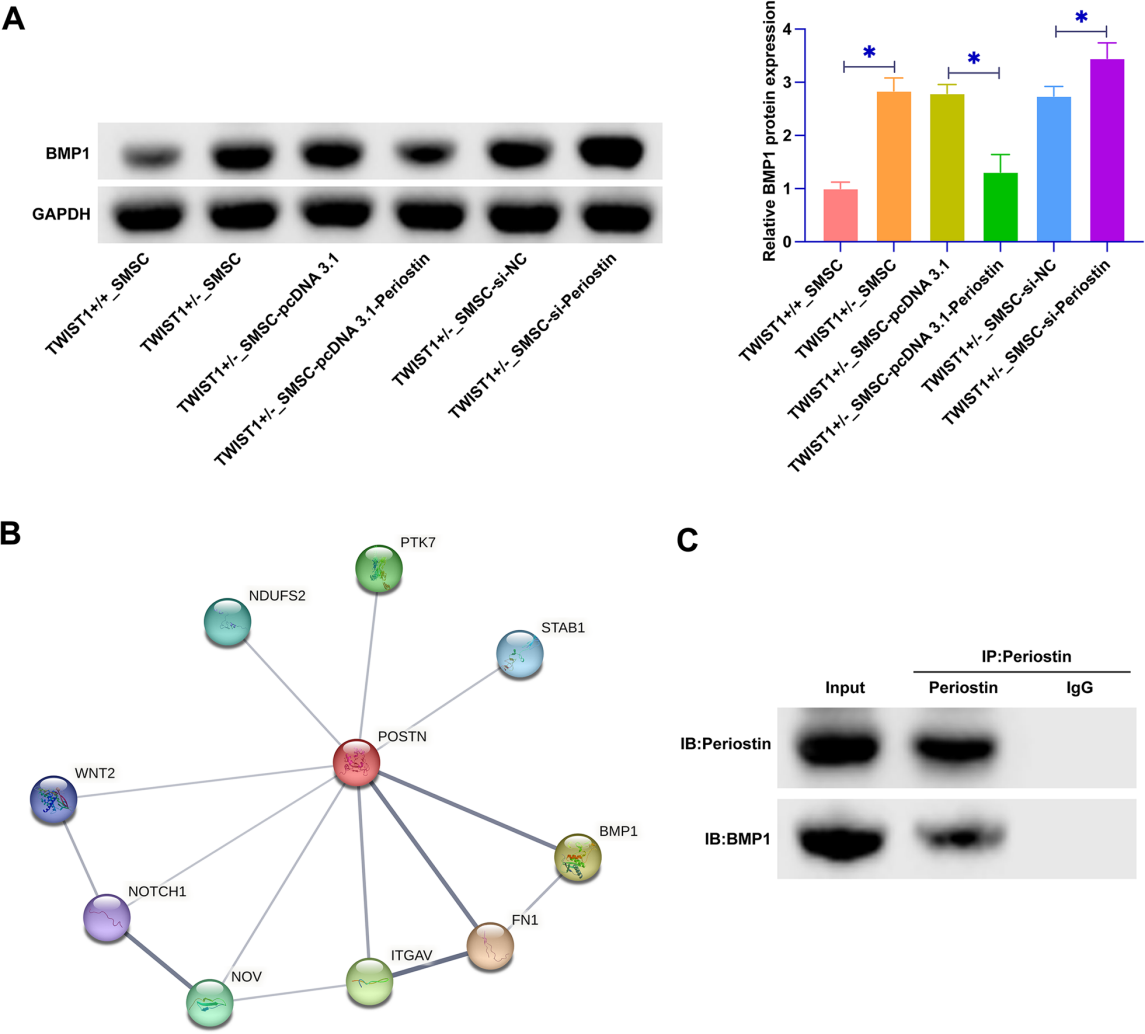
Periostin regulates BMP1 expression in SMSCs. **A** Western blot assays were performed to evaluate the protein expression of BMP1 in SMSCs across the different groups. **B** The interactive network between Periostin and BMP1 was investigated using the bioinformatics website https://cn.string-db.org. **C** CO-IP experiments were conducted to detect the interaction between Periostin and BMP1. Data were represented as mean ± SD (N = 3). *P < 0.05

We next examined the impact of Periostin on BMP1 protein expression. BMP1 expression in TWIST1^+/−^ SMSCs was elevated compared to that in TWIST1^+/+^ SMSCs. Notably, Periostin overexpression led to a significant reduction in BMP1 protein levels, whereas Periostin knockdown exhibited the opposite effect, thereby indicating a regulatory relationship (Fig. 4A). We employed bioinformatics tools to explore Periostin's protein interaction network. This analysis revealed a potential interactive relationship between Periostin and BMP1 (Fig. 4B). This interaction was confirmed through CO-IP experiments (Fig. 4C). These findings collectively elucidated that Periostin modulated BMP1 expression in SMSCs. This insight not only unveiled a novel regulatory axis within the cellular milieu of craniosynostosis but also accentuated the intricate molecular dialogue between Periostin and BMP1.

### Knockdown of BMP1 inhibits the proliferation and osteogenic differentiation of SMSCs

**Fig. 5:**
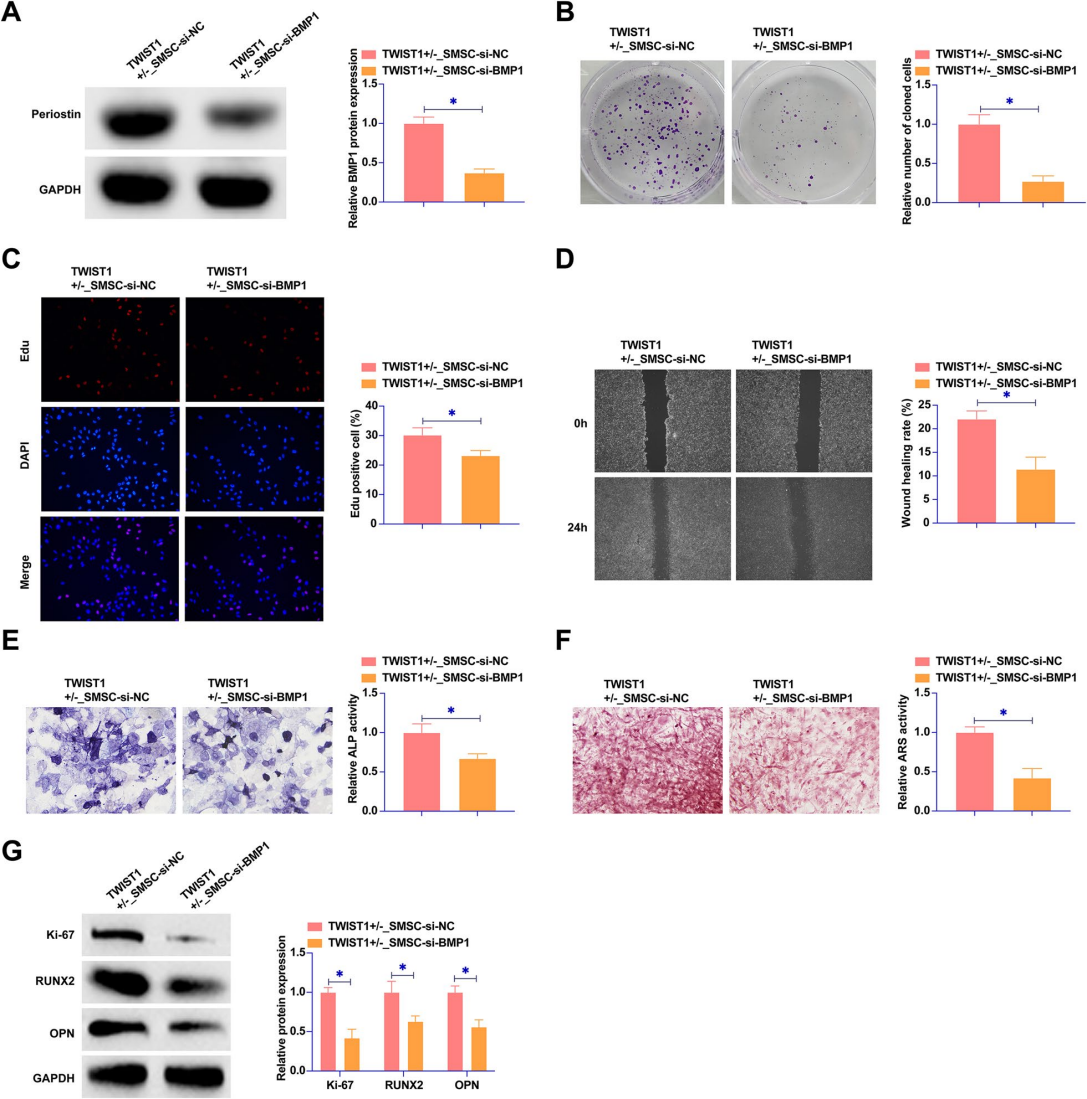
Knockdown of BMP1 inhibits the proliferation and osteogenic differentiation of SMSCs. siRNA targeting BMP1 was transfected into the SMSCs of the TWIST1^+/−^ group. **A** Western blot assays were utilized to assess BMP1 protein expression in SMSCs across the different groups. **B** Colony formation assays were conducted to evaluate the proliferative capacity of SMSCs. **C** EdU assays were used to further quantify the proliferation of SMSCs. **D** Wound healing assays were carried out to measure the wound closure rate of SMSCs. **E** ALP staining was employed to determine the ALP activity in SMSCs. **F** ARS staining was used to observe calcium deposition in SMSCs. **G** Western blot analysis was conducted to assess the expression levels of Ki-67, RUNX2, and OPN in SMSCs. Data were represented as mean ± SD (N = 3). *P < 0.05

We next focused on elucidating the role of BMP1 in SMSCs by transfecting TWIST1^+/−^ SMSCs with siRNA targeting BMP1. Figure 5A demonstrated the effectiveness of this approach, with a marked reduction in BMP1 protein levels within SMSCs post-transfection. BMP1 knockdown on cellular functions was then thoroughly investigated. Colony formation and EdU assays revealed a significant decrease in both the clonal growth potential and the proportion of EdU-positive cells in SMSCs (Fig. 5B, C). Moreover, wound healing assay (Fig. 5D) highlighted a reduced healing rate in SMSCs post-BMP1 silencing. ALP and ARS staining further corroborated these findings by indicating reduced ALP activity and calcium deposition in SMSCs following BMP1 knockdown (Fig. 5E, F). In addition, a notable suppression in the expression of key proliferation and differentiation markers, including Ki-67, RUNX2, and OPN, was observed in SMSCs post-BMP1 silencing (Fig. 5G). Collectively, these results underscored the critical role of BMP1 in regulating both the proliferative and osteogenic differentiation capacities of SMSCs.

### The Periostin/BMP1 axis is a crucial pathway for regulating the proliferation and osteogenic differentiation of SMSCs

**Fig. 6:**
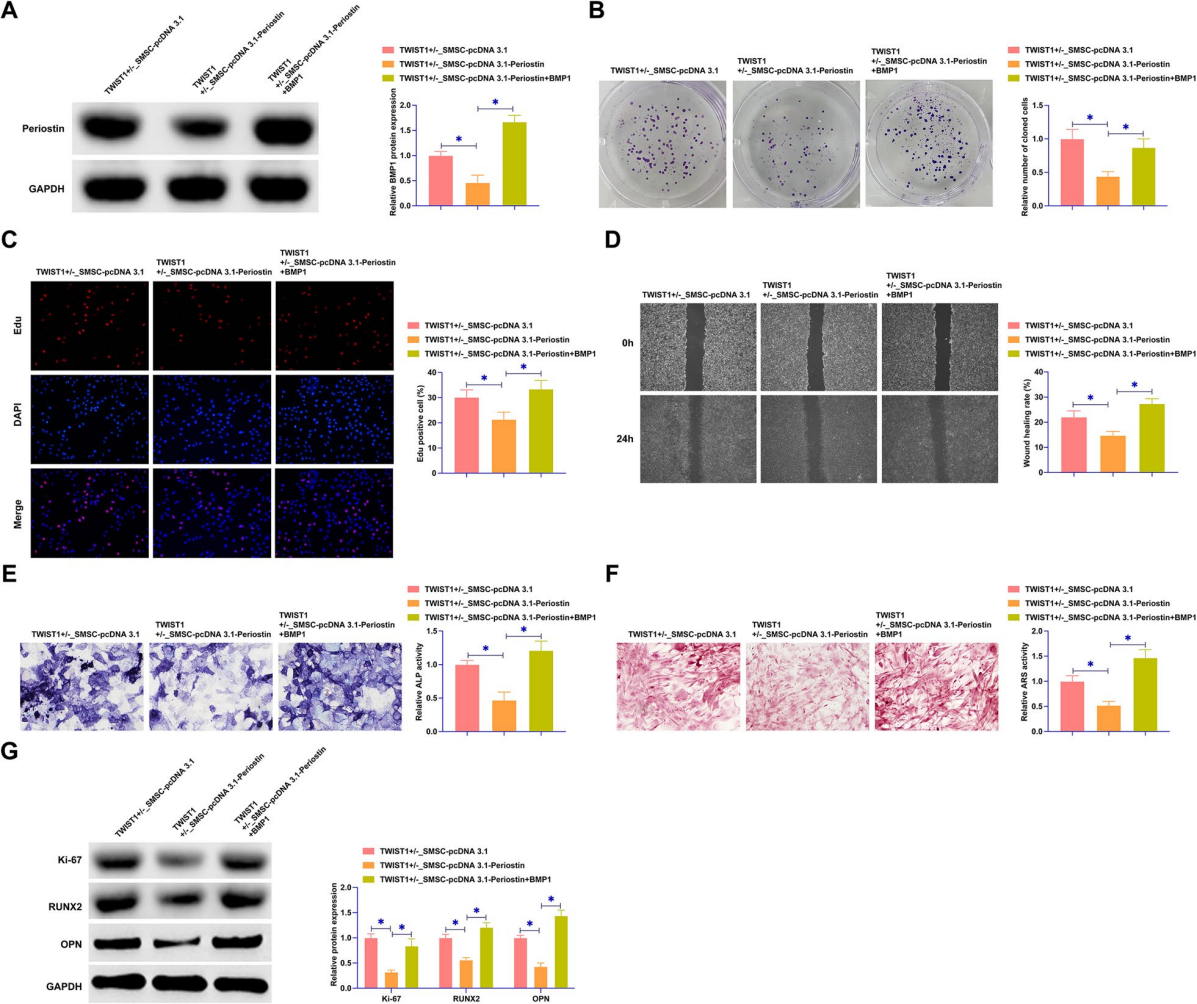
Periostin/BMP1 axis regulates SMSC proliferation and osteogenesis. pcDNA 3.1-Periostin and pcDNA 3.1-BMP1 were co-transfected into the SMSCs of the TWIST1^+/−^ group. **A** Western blot assays were conducted to measure BMP1 protein expression in the SMSCs across the groups. **B** Colony formation assays were performed to evaluate the proliferative capacity of SMSCs. **C** EdU assays were utilized to further quantify the proliferation of SMSCs. **D** Wound healing assays were carried out to assess the wound closure rate of SMSCs. **E** ALP staining was employed to determine the ALP activity in SMSCs. **F** ARS staining was used to observe calcium deposition in SMSCs. **G** Western blot analysis was conducted to measure the expression levels of Ki-67, RUNX2, and OPN in SMSCs. Data were represented as mean ± SD (N = 3). *P < 0.05

We co-transfected TWIST1^+/−^ SMSCs with pcDNA 3.1-Periostin and pcDNA 3.1-BMP1. The results intriguingly revealed that the inhibitory effect of pcDNA 3.1-Periostin on BMP1 expression was effectively counteracted by the co-expression of pcDNA 3.1-BMP1 (Fig. 6A). Colony formation and EdU assays indicated that while overexpression of Periostin suppressed the clonogenic capabilities and EdU-positive cell proportion in SMSCs, this suppression was mitigated upon BMP1 overexpression (Fig. 6B, C). Furthermore, the wound healing assay (Fig. 6D) demonstrated that the inhibitory impact of Periostin overexpression on SMSC wound healing rate was reversed with the concurrent overexpression of BMP1. This interplay was further explored in ALP and ARS staining. Downregulation of ALP activity and calcium deposition due to Periostin overexpression was effectively counteracted by BMP1 overexpression (Fig. 6E, F). Additionally, the protein expression profiles of key markers such as Ki-67, RUNX2, and OPN, which were downregulated by Periostin overexpression, exhibited a reversal in this trend upon BMP1 overexpression (Fig. 6G). These comprehensive data highlighted a regulatory mechanism wherein Periostin modulates SMSC proliferation and osteogenic differentiation through its influence on BMP1 expression.

## Discussion

This study explored the Periostin/BMP1 axis in regulating SMSC proliferation and osteogenic differentiation and revealed its effect on craniosynostosis in TWIST1^+/−^ mice. The Periostin/BMP1 axis was involved in regulating the proliferation and osteogenic differentiation of SMSCs in TWIST1^+/−^ mice, and targeting the Periostin/BMP1 axis repaired craniosynostosis in TWIST1^+/−^ mice, providing an SMSC-based treatment strategy for craniosynostosis with potentially beneficial implications for clinical practice.

Periostin can exert functionally in the initial activation of skeletal stem cells in early repair, the active phase of cartilage and bone deposition in fracture callus, and the final phase of bone bridging and stem cell reconstruction [15]. Injection of recombinant mouse Periostin could effectively improve suture closure in TWIST1^+/−^ mice and inhibit suture cell proliferation and osteogenic differentiation [7]. Moreover, micro-nano morphology promoted osteogenic differentiation of bone MSCs by mediating Periostin expression [16]. This study also found that over-expressing periostin improved craniosynostosis in TWIST1^+/−^ mice. Meanwhile, periostin overexpression significantly inhibited the proliferation and osteogenic differentiation of SMSCs isolated from TWIST1^+/−^ mice, but the effects on other sutured cells were unclear. MSCs have shown great potential in cranial suture regeneration and repair of malformation of the skull [17].

Further, periostin could target and regulate BMP1, and there was a direct interaction between them. This was consistent with previous findings [18]. However, it was not clear how periostin directly affects BMP1 protein expression. Therefore, it is necessary to explore whether periostin regulates BMP1 protein expression by affecting the transcriptional regulation or protein stability of BMP1 in subsequent studies. BMP1 could promote the osteogenic differentiation process of MSCs [19]. This phenomenon was also found in this study, showing that BMP1 increased ALP, RUNX2, and OPN during osteogenic differentiation of SMSCs, and promoted calcium deposition. Therefore, reducing the osteogenic differentiation process of SMSCs may prevent accelerated growth of frontal bone and parietal bone [20], which will help improve craniosynostosis.

Although our findings showed a regulatory role of the Periostin/BMP1 axis in both mouse models and SMSC models for craniosynostosis, we need to further correlate these findings with clinical data related to human diseases. Conducting broader population studies, including analyzing samples from patients with craniosynostosis, could better validate the Periostin/BMP1 axis. In addition, understanding the specific signal transduction pathways and downstream targets in which Periostin/BMP1 regulates coronary craniosynostosis is a potential direction for further research. Further experiments in molecular and cell biology can help reveal the interaction of the Periostin/BMP1 axis with other signaling pathways. New drug therapeutic strategies targeting the Periostin/BMP1 axis may be considered, but the potential of this drug development requires further experimental validation and clinical trials in future studies.

In short words, this study revealed Periostin/BMP1 axis’s role in coronary craniosynostosis in TWIST1^+/−^ mice by deeply exploring SMSC proliferation and osteogenic differentiation. This discovery of modulating SMSCs by targeting periostin will help improve future therapeutic interventions for craniosynostosis.

## Data Availability

The datasets used and/or analyzed during the present study are available from the corresponding author upon reasonable request.
